# Subspine femoroacetabular impingement: retrospective study of a series of patients treated by hip arthroscopic resection

**DOI:** 10.1007/s00402-022-04761-2

**Published:** 2023-02-08

**Authors:** Alberto Frances Borrego, Alvaro Martinez Garcia, Laura Del Baño Barragán, Alberto Rodríguez González, Marta Echevarría Marín, Fernando Marco Martinez

**Affiliations:** Hospital Clínico San Carlos, Profesor Martin Lagos s/n, 28040 Madrid, Spain

**Keywords:** Femoroacetabular impigement, Hip, Subspine impigement, Hip arthroscopy

## Abstract

**Background:**

Femoroacetabular impingement syndrome (FAIS) is a common hip pathology that causes pain and functional limitation in young patients. subspine femoroacetabular impingement (SFAI) is an increasingly diagnosed extra-articular subtype that occurs from mechanical conflict of the anteroinferior iliac spine (AIIS) with the cervico-diaphyseal junction during hip flexion, which is poorly described in the literature.

**Questions/purposes:**

We aimed to describe the clinical, functional, and radiological results of the arthroscopic treatment of a group of patients with SFAI treated in our Hip Unit.

**Study design:**

Case series.

**Methods:**

We present a retrospective study of ten patients with SFAI treated between 2013 and 2020 with arthroscopic resection. Clinical results were assessed with scales such as visual analog scale (VAS); modified Harris Hip Score (mHHS), and Hip disability and Osteoarthritis Outcome Score (HOOS). Radiological results were assessed with radiological measurements, magnetic resonance imaging (MRI), and computed tomography (CT) reconstructions.

**Results:**

Six patients had a Type III AIIS and four of them had Type II. Two patients had previously been surgically treated for FAIS. The range of motion improved in flexion from 107 ± 11 degrees before surgery to 127.5 ± 6 degrees (*p* = 0.005). MHHS improved from 48.1 (38–75.3) before surgery to 83.1 (57–91) (*p* = 0.007) and HOOS improved from 65.2 (58–75) to 89 (68.1–100) (*p* = 0.007). VAS improved from 7.3 (5–9) pre-surgical to 2.5 (0–8) post-surgical (*p* = 0.005). We did not have significant complications except for an asymptomatic case of heterotopic ossification (Brooker I).

**Conclusion:**

Arthroscopic decompression of AIIS in SFAI patients is a safe procedure that provides satisfactory short-term functional results, improving clinical symptoms, function, sports performance, and range of motion in our study.

## Introduction

Femoroacetabular impingement syndrome (FAIS) is a common cause of pain and disability in the young adult’s hip due to impingement or abnormal contact between the proximal femoral neck and the acetabulum resulting in pain with hip flexion, internal rotation, and other flexion-based cutting and pivoting activities. There are two large groups of FAIS recognized, the intra-articular and the extra-articular impingement. The intra-articular FAIS-pincer and cam types are the most common. With the use of new imaging techniques and anthropometric measurements, other pathologies have been included in FAIS. Extra-articular FAIS is being recognized as an important cause of clinical symptoms caused by a prominent AIIS. This is an anatomical deformity that can cause pain and limited range of motion in patients with no previous hip symptoms and patients who have already undergone a previous hip arthroscopy that was misdiagnosed with other types of FAIS [1, 2]. Subspine femoroacetabular impingement (SFAI) may appear isolated or associated with other types of intra-articular or extra-articular lesions. The increase in its incidence/diagnosis seems to be due to better knowledge of the anatomical deformity of the AIIS and a better understanding of the kinematics of the hip joint [3–7]. This morphological abnormality of the AIIS can be a subsidiary of surgical treatment by resection to avoid mechanical conflict [3, 8–11]. Despite the literature, many questions regarding the prevalence, existence, and indications for decompression of AIIS should be considered. Our purpose is to analyze the results of SFAI arthroscopically treated in 11 patients analyzing surgical technique, radiological, clinical, and functional results. We hypothesized in our preliminary experience that SFAI arthroscopically treated yields good short-term results.

## Materials and methods

**Fig. 1:**
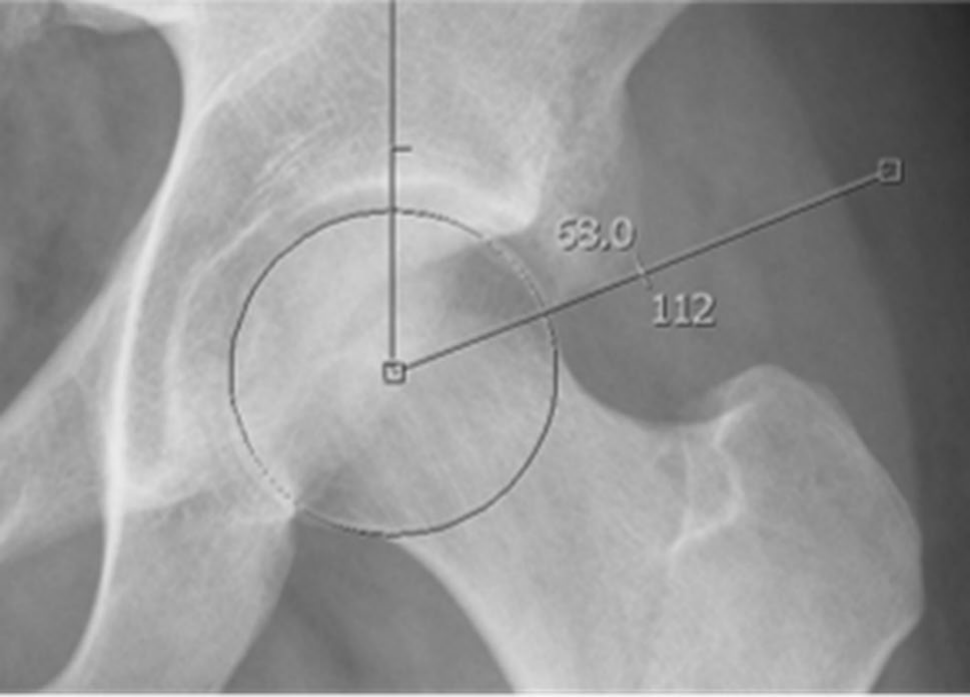
Center-subspine angle

We conducted a retrospective study of 11 patients arthroscopically treated between 2013 and 2020, approved by the institution review board of our center. Those patients were included from our series of 248 patient arthroscopically treated for hip pathology in those 8 years. Inclusion criteria were diagnosis of SFAI previous to the surgery, minimum follow-up of 6 months, and positive consent to participate in this study. One patient was excluded from the original series because of a psychiatric disorder and seeking workers’ compensation, being unable to report appropriately in the functional scales. All the patients went through a conservative treatment with no satisfactory improvement before undergoing surgery. The rest (ten patients) were included in this study. All patients were surgically treated by one single surgeon specialized in hip arthroscopy. Medical records were reviewed by two independent observers out of the Hip Unit for epidemiological data: age, gender, medical history, history of previous hip surgery, job occupation, and sport activity level (Tegner activity level scale). Preoperative and postoperative clinical data were collected: hip exploratory maneuvers, hip joint balance assed with a digital goniometer, visual analog scale (VAS), patient satisfaction and clinical scales, the modified Harris Hip Score (mHHS), and the Hip disability and Osteoarthritis Outcome Score (HOOS). A preoperative and postoperative radiological study was conducted: plain radiology, computed tomography (CT) (2D and 3D), and magnetic resonance imaging (MRI). Radiological measurements were measured in anteroposterior pelvis view radiography and late Dunn hip joint (dunn) view radiography: alpha angle, center–edge angle in the anteroposterior view, center-subspine angle (an angle between a line perpendicular to the floor through the head center and a line passing through the edge of the AIIS) (Fig. 1), acetabular version [7, 12], and the classification of the prominence of the AIIS in three grades of increasing magnitudes proposed by Hetsroni et al. [4]. The follow-up time of all patients and the occurrence of postoperative complications were recorded. A statistical study of the data obtained with the SPSS program (IBM SPSS Statistics for Windows, Version 26.0. Armonk, NY: IBM Corp) was carried out, comparing the quantitative variables with a non-parametric test (Wilcoxon test).

**Fig. 2:**
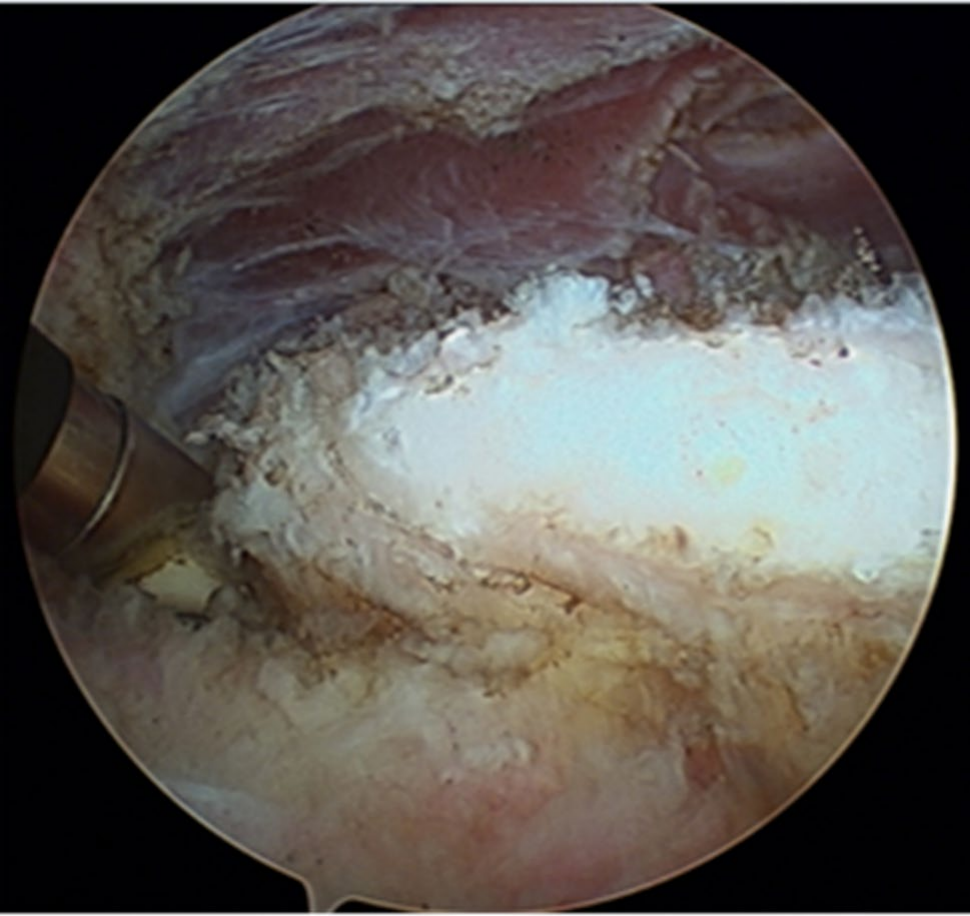
Anterior inferior iliac spine (AIIS) approach

**Fig. 3:**
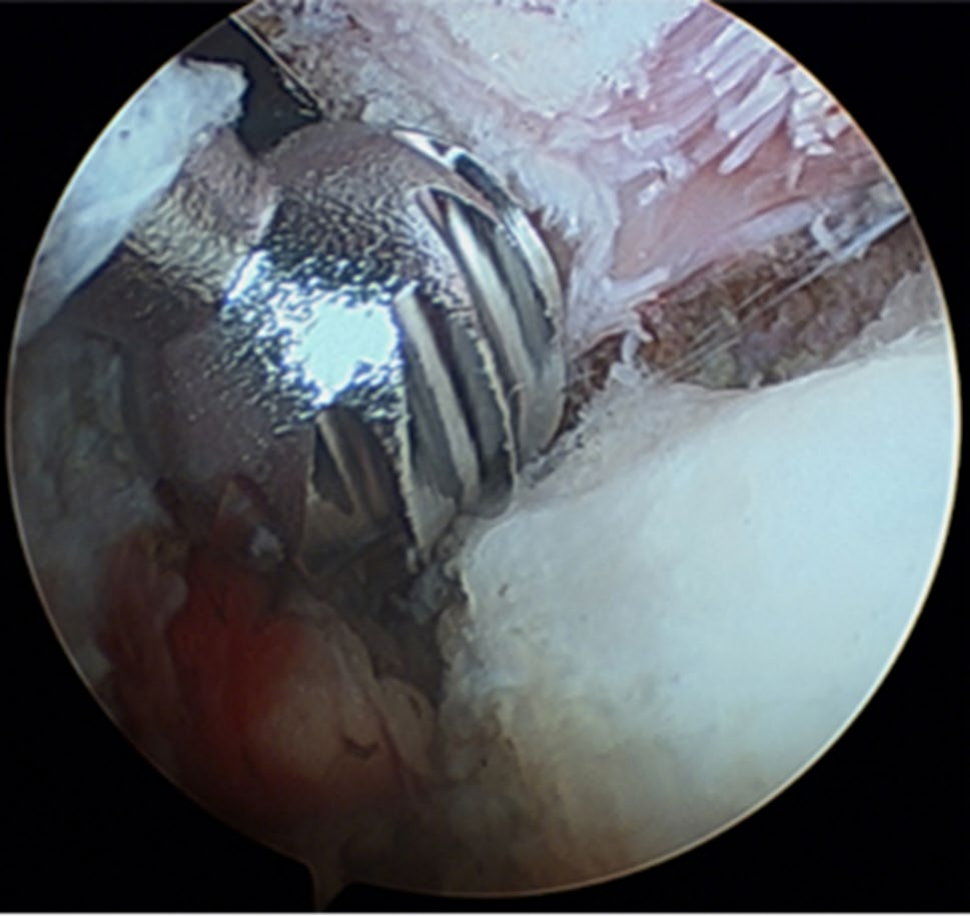
Anterior inferior iliac spine (AIIS) resection

Patients were treated with two different surgical techniques, depending on the associated lesions. In the first group, in patients with primary intra-articular or extra-articular joint-associated lesions—cam deformity or associated pincer—or secondary labral or chondral lesion, standard arthroscopic treatment was performed (inside-out). A lateral decubitus standard hip arthroscopy was performed for the surgical treatment of associated lesions. After central compartment work was done, the AIIS was approached through an inside-out capsulotomy, and the capsule-labral space was developed, to proceed with the dissection and resection with a burr under fluoroscopy with the help of an anterior portal for better access. In the second group, patients with no associated lesions or with lesions already treated in previous arthroscopy procedures, direct extra-articular arthroscopy was performed over the AIIS. Under fluoroscopy control, the AIIS was located with two long trocars, one for vision and another working portal to proceed with the resection, being the rectus tendon preserved (Figs. 2 and 3). We used anteroposterior and lateral (Dunn) views for the quantification of the amount of resection done during surgery.

**Fig. 4:**
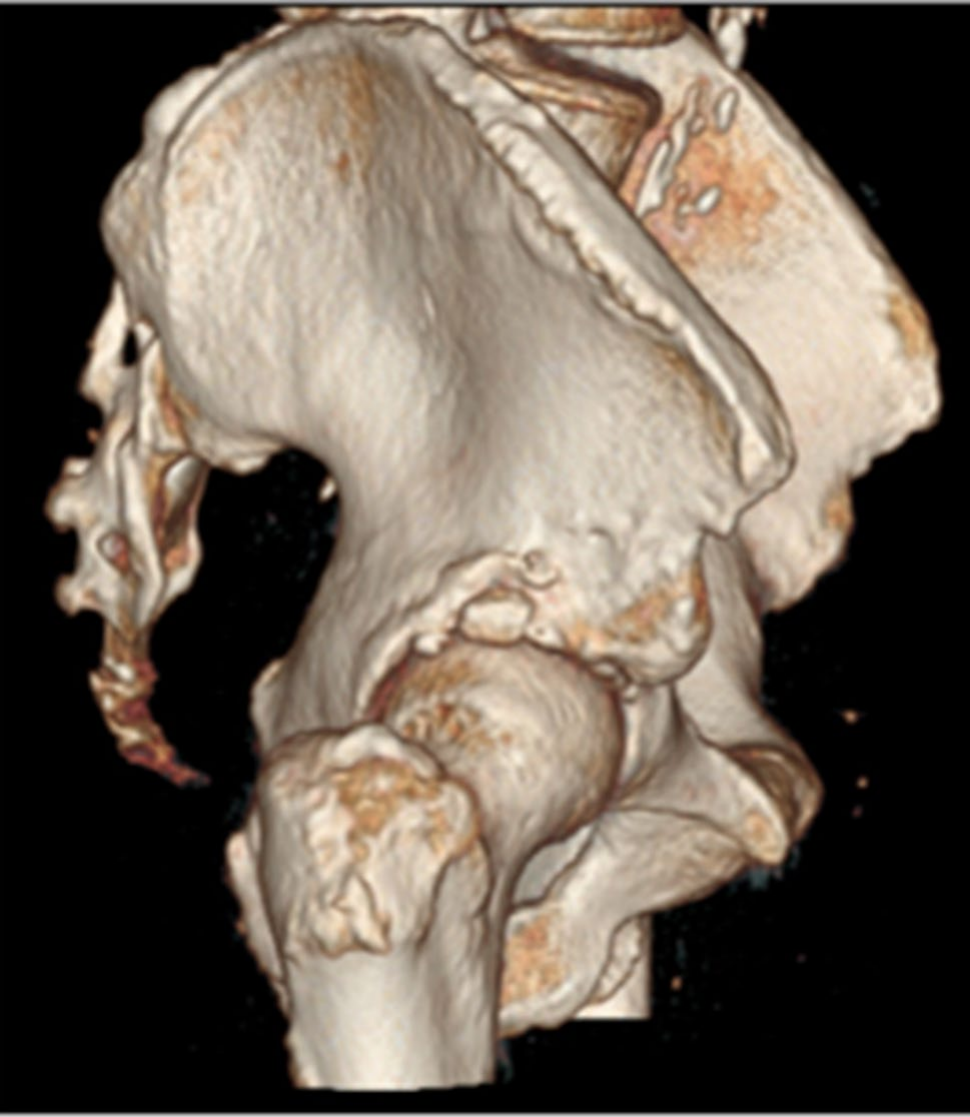
Antero-posterior and axial views—3D

**Fig. 5:**
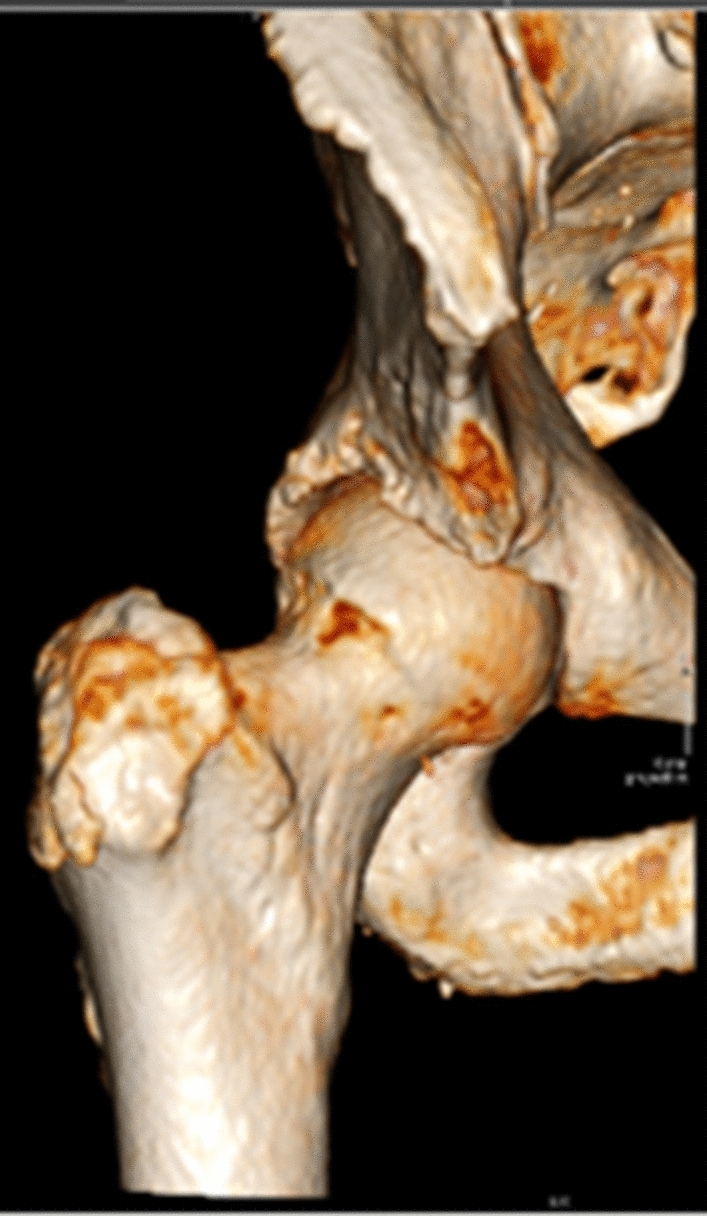
3D computed tomography (CT)

All patients were treated in a Major Ambulatory Surgery Unit and were discharged the same day. Postoperative treatment was performed and included analgesic control according to needs, celecoxib 200 mg for 1 month, enoxaparin 40 mg subcutaneously for 15 days, and partial weight bearing according to tolerance for patients without associated lesions and no weight bearing when treatment of associated lesions was required (labral suture, cartilage microfracture technique). The postoperative follow-up protocol included radiological anteroposterior and lateral views of 2D and 3D CT (Figs. 4 and 5) (weeks 1–3–12–24 and then annually till the end of the follow-up period).

## Results

Our series included ten patients (five women and five men), with an average age of 33 years (25–59) with eight of the ten patients under the age of 35 years (Table 1). All patients had a sedentary job, without extreme physical efforts with loads. Two of the patients went previously through hip arthroscopy because of mixed-type intra-articular FAIS without improvement of symptoms after the mean surgery. The average follow-up was 34 months (14–74).

**Table 1:** Epidemiologic data (BMI: body mass index)

No	Age	Sex	Work	Body mass index	Sport	Previous surgery
1	26	Woman	Clerk	21.3	Running	No
2	59	Man	Office	27.4	Horse riding	No
3	28	Man	Office	23.4	Football	No
4	33	Man	Computer	24.8	Bike	No
5	45	Woman	Accounting	28.5	Pilates	No
6	27	Woman	Office	20.2	Bike	No
7	27	Woman	Office	23.7	Gym	No
8	25	Woman	Cook	19.5	Bike	Femoroplasty and labral repair
9	31	Man	Office	27.3	Basketball	No
10	26	Man	Office	21.9	Swimming	Femoroplasty and labral repair

Postoperative results were evaluated in our office with a minimum follow-up of 6 months. The flexion, and internal and external rotation improves, although no statistical significance was found. The functional scales and the VAS scale improve with statistical significance and, in terms of satisfaction, all the patients were “very” (4/5) or “pretty” (5/5) satisfied on a scale out of 5. (Tables 2, 3, 4 and 5).

**Table 2:** Preoperative and postoperative range of motion per patient (hip flexion—preoperative, hip flexion—postoperative, hip internal rotation—preoperative, hip internal rotation—postoperative, hip external rotation—preoperative, hip external rotation—postoperative)

No	Hip flexion preoperative	Hip flexion postoperative	Hip internal rotation preoperative	Hip internal rotation postoperative	Hip external rotation preoperative	Hip external rotation postoperative
1	100	110	20	20	30	40
2	90	130	15	40	25	40
3	120	130	40	40	40	40
4	120	130	40	40	40	40
5	120	130	20	30	30	40
6	110	130	30	40	30	40
7	110	130	20	40	20	40
8	110	130	20	30	40	40
9	100	130	10	40	40	40
10	90	125	10	15	15	40

**Table 3:** Functional and VAS scales per patient

No	Visual analog scales preoperative	Visual analog scales postoperative	Modified Harris Hip score preoperative	Modified Harris Hip score postoperative	HOOS Hip score preoperative	HOOS Hip score postoperative
1	9	8	48	57	63.7	68.1
2	9	0	53	91	66.9	100
3	6	1	48	87	73.8	96.9
4	8	0	48	91	58.1	91.3
5	9	5	46	74	56.3	83.1
6	5	2	45	85	53.3	82.3
7	6	1	49	83	67.1	86.3
8	6	0	48	90	73	96
9	6	3	48	90	75	98
10	9	5	38	71	51.2	85.5

**Table 4:** Range of motion and functional scales

	Preoperative	Postoperative	*p*
Flexion	107 ± 11	127.5 ± 6	0.005
Internal rotation	22.5 ± 6	33.5 ± 10.3	0.017
External rotation	31 ± 8.9	40 ± 8.6	0.026
mHHS	48.1 ± 2.5	83.3 ± 12.1	0.007
HOOS	65.2 ± 7.2	89 ± 10.6	0.007
VAS	7.3 ± 2.6	2.5 ± 1.6	0.003

**Table 5:** Tegner scale per patient

No	Tegner preoperative	Tegner postoperative
1	6	6
2	5	5
3	6	6
4	3	3
5	4	4
6	6	6
7	4	4
8	7	7

All patients performed a sporting activity with a Tegner scale greater than 3 in all cases before the surgery and this activity sport was maintained at the same level after the surgery.

No significant complications were reported except for an asymptomatic heterotopic ossification case (Brooker 1), none of the patients need reintervention at the end of the follow-up.

**Fig. 6:**
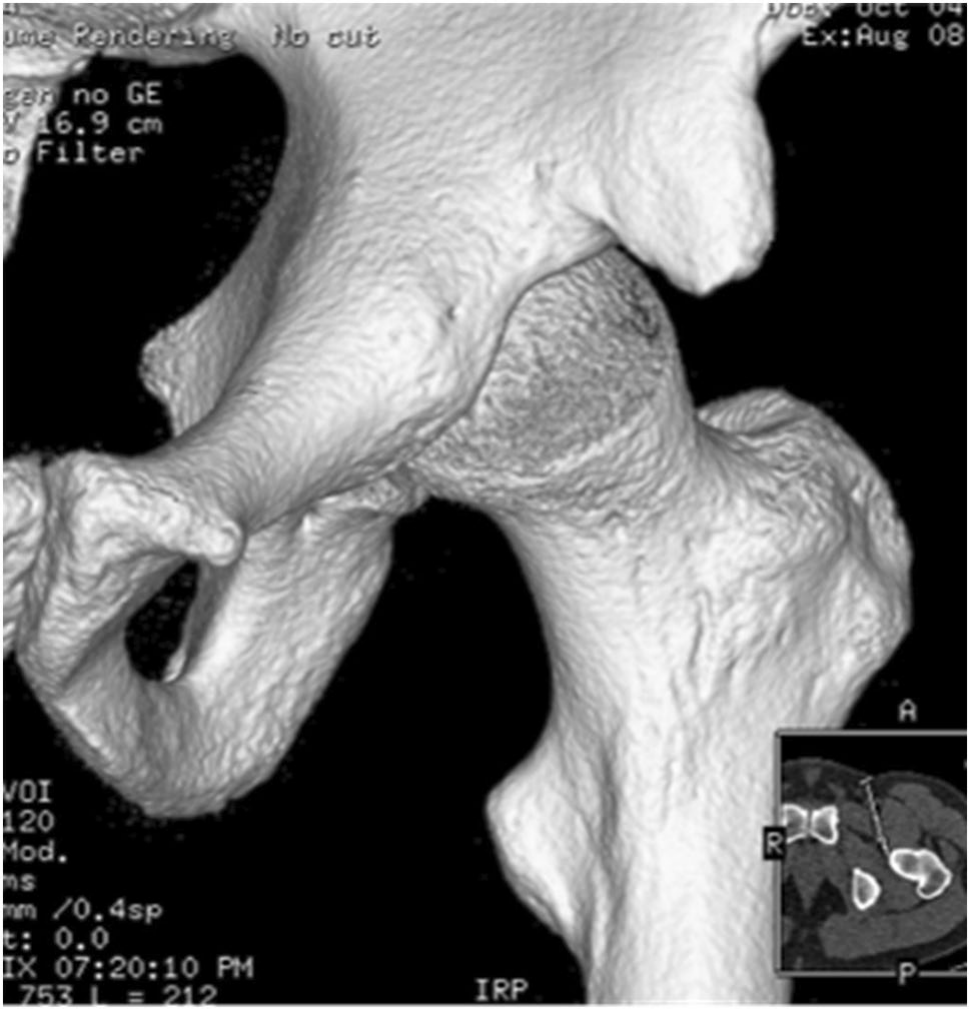
Type III anterior inferior iliac spine morphology

**Fig. 7:**
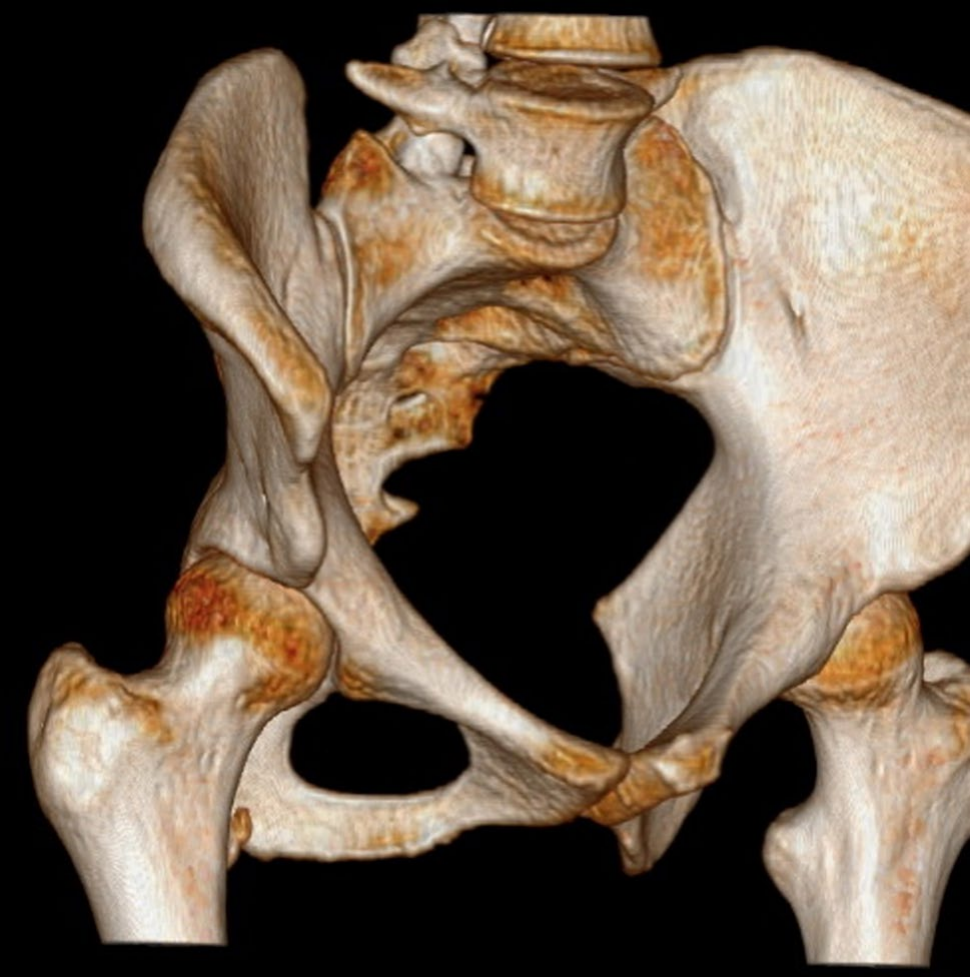
Type II anterior inferior iliac spine morphology

In terms of the radiological results, six of the ten patients had an AIIS morphology Type III, where the prominence of the AIIS exceeded the acetabular edge (Fig. 6), and four patients had Type II, where the AIIS reached the spine to the acetabular edge (Fig. 7). None of the patients had Type I. The only radiological parameter changing was the center-subspine angle moving from 46.6º to 37.1º (*p* = 0.17). (Tables 6 and 7).

**Table 6:** Radiological measurements per patient

No	Preoperative center-edge	Postoperative center-edge	Preoperative center-subspine	Postoperative center-subspine	Acetabular version	Alpha angle
1	27.5	41.4	43.7	27.1	41.5	8
2	38.4	32.2	42.1	46.3	42.4	19.0
3	41	39.4	63.7	36.2	38.9	17.3
4	38	36.4	42.1	39.8	24	18.1
5	47.3	36.7	52.9	35.2	57	13.6
6	26	26	42.2	31.4	38.3	16.9
7	45.9	34.5	35.1	36.6	42.9	17.7
8	41.4	38.5	45	39.1	40	15.5
9	40	35.5	45	38.2	40	15.6
10	43	35.2	54.1	41	52.7	11.2

**Table 7:** Radiological measurements

	Preoperative	Postoperative
Acetabular center-edge	38.8	35.2
Center-subspine angle	46.6	37.1
Alpha angle	41.8	41.8
Acetabular version	15.1	15.1

## Discussion

The results found in the literature after surgical treatment of intra-articular FAIS—cam and pincer morphology yield good outcomes [13, 14]. Unfavorable outcomes after treatment have led us to understand other unrecognized causes of impingement that can appear with or without these intra-articular conflicts. Some of them are related to extra-articular FAIS—iliopsoas, ischiofemoral, pelvitrocanteric, and subspine impingement [3, 15]. Multiple studies support that different morphologies of AIIS and the subspinal region can produce mechanical conflicts between the femoral neck region and the acetabulum [4, 12, 16–19]. Different causes have been described for this aberrant morphology of the AIIS, like avulsion in young athletes of the proximal head of the rectus femoral muscle [20]; traction hypertrophy as a result from the repeated strain placed across the AIIS during running, cutting, and kicking sports during the adolescent period; but in other cases, the etiology is not clear. Regarding the surgical treatment of this pathology, there are not many reports in the literature, composed mainly of case reports or small series of patients [3, 8–10].

We have used like most studies, X-rays (AP, lateral, false profile) [21] and CT, to evaluate AIIS morphology. Some suggest the use of ultrasound examination with good results [22] and MRI 3D [21]. Nevertheless, it appears 3D CT is better to classify the morphology of the AIIS [23] and actual trends are working with dynamic studies with CT to improve diagnostic accuracy [11, 24].

Open decompression was first described by the anterior hip approach, although AIIS decompression arthroscopic techniques are the most used techniques nowadays [9, 10]. In our patients, we have selected an inside-out technique in cases with intra-articular lesions (cam, labral or chondral damage) but in the rest of the patients, where isolated SFAI was found, we went for a direct approach to the AIIS with an outside-in technique guided by fluoroscopy. By doing that, we avoided the damage to the IFL observed with the intraportal capsulotomy. It is known that tissue damage at the anterior capsule was observed after AIIS trimming [3]. The width of the proximal capsular attachment is 5 mm. Authors are aware of potential damage to the capsule and pericapsular structures when using a transverse intraportal capsulotomy for arthroscopic AIIS decompression [25]. It is recommended to do a transverse intraportal capsulotomy at least 5–10 mm from the tip of the labrum [26]. The technique is a safe procedure and only one of our patients had heterotopic ossification (Brooker 1) with no symptoms regardless of prophylaxis with celecoxib. One of the complications described in the literature, the disinsertion of the head of the rectus femoral muscle [27], is probably the result of very aggressive resection of the AIIS, and avoidable with the proper surgical technique, since we do not find this complication in our series.

In this study, we found that complete arthroscopic resection of a prominent AIIS Type III (six cases) and Type II (four cases) in preoperative symptomatic patients resulted in functional improvement after surgery. All of our patients obtained clinically significant outcomes on the VAS scale, mHHS, and HOOS with an average follow-up of 28 months (8–68). The ten patients were “very” or “pretty” satisfied, in accordance with the previous literature. Results described in the literature are very satisfactory and few complications have been described, so although the evidence is poor, arthroscopic subspine decompression could become in the future the gold standard in this pathology [3, 11, 28].

All our patients began with symptoms when doing sports. Before beginning with pain, the preoperative Tegner level average was 5.3 (competitive sports cycling, cross-country skiing, or recreational jogging) and, after surgery, at 12 months of evaluation, all of them had regained their previous sport level. Sports or extreme range-of-motion activities can be involved in the development of pain as motion and hip impingement appears [10, 17]. Hyper-flexible athletes have a higher prevalence of SIS with a modest rate of return to sport and good-to-excellent patient-reported outcomes [29], more related to kinematics than with AIIS deformity. We should understand the difference between morphology and impingement. Authors pointed out that SFAI has been associated with high range-of-motion activities with different AIIS morphologies. We can face two scenarios: patients with relatively small AIIS could impinge with an extended range of motion, and patients in front of non-impinging Type II or III AIIS in patients with a short range of motion in their hips. Authors show in dynamic computed tomographic imaging models where 23.7% of the hips had impingement between the femur and AIIS but greater than 50% of these cases were associated with a relatively normal Type I AIIS [30]. However, most authors think that an altered AIIS morphology is directly related to the probability of subspinal impingement, and the amount of the deformity is important as all our patients had Type II or III AIIS [5, 17]. In our study, we found that all of our patients increased their range of motion (flexion, internal rotation, and external rotation) and all of them obtained clinically significant outcomes in the VAS scale and all functional scales due to hips that do not impinge anymore.

Extra-articular hip impingement refers to a wide spectrum of non-intra-articular disorders other than FAIS [31]. Among them, SFAI should be kept in mind while preoperative diagnosis is done in patients with hip pain [32]. In this study, we included two patients out of ten who had been operated on before because of cam FAIS with an unfavorable result after surgery with a non-recognized SFAI. Authors found 24% of patients with intra-articular FAIS and SFAI associated [6]. For this reason, some surgeons routinely perform subspine decompressions during their arthroscopies, and others do not [26]. So, we should be looking for extra-articular impingement in patients with intra-articular FAIS in order not to fail in our results [32]. It is also known that deformities related to femoral anteversion can increase the rate of impingement. Anterior extra- and intra-articular hip impingement can be present in patients who have FAIS with decreased femoral anteversion [24].

The limitations in our study were that it was a retrospective study with a small number of patients with short-term follow-up. Furthermore, clinical scores were not measured preoperatively and patients were addressed in two different arthroscopic techniques (inside-out and extra-articular technique) so potentially additional pathology could be missed. Nevertheless, our results, in accordance with the literature, support arthroscopic decompression in the subspine impingement as a safe technique with good clinical and functional medium terms results.

The surgical indication is the key to success and should be based on symptoms, clinical assessment, X-ray and CT images, and, overall, in a customized kinematics understanding of each patient’s hip.

Our findings are of clinical relevance and match other published literature on this subject.

Future considerations: Advanced CT-based kinematics models of this same group of patients will lead us to understand how their hips improved after surgery in terms of impingement clearance.

## Conclusion

Arthroscopic decompression of AIIS in SFAI patients is a safe procedure that provides satisfactory short-term functional results, improving clinical symptoms, function, sports performance, and range of motion in our study.
